# Function Investigations and Applications of Membrane Proteins on Artificial Lipid Membranes

**DOI:** 10.3390/ijms24087231

**Published:** 2023-04-13

**Authors:** Toshiyuki Tosaka, Koki Kamiya

**Affiliations:** Division of Molecular Science, Graduate School of Science and Technology, Gunma University, Gunma 376-8515, Japan

**Keywords:** membrane proteins, ion channels, nanopores, giant lipid vesicles, liposome, proteoliposome, planar bilayer lipid membrane, artificial cell model

## Abstract

Membrane proteins play an important role in key cellular functions, such as signal transduction, apoptosis, and metabolism. Therefore, structural and functional studies of these proteins are essential in fields such as fundamental biology, medical science, pharmacology, biotechnology, and bioengineering. However, observing the precise elemental reactions and structures of membrane proteins is difficult, despite their functioning through interactions with various biomolecules in living cells. To investigate these properties, methodologies have been developed to study the functions of membrane proteins that have been purified from biological cells. In this paper, we introduce various methods for creating liposomes or lipid vesicles, from conventional to recent approaches, as well as techniques for reconstituting membrane proteins into artificial membranes. We also cover the different types of artificial membranes that can be used to observe the functions of reconstituted membrane proteins, including their structure, number of transmembrane domains, and functional type. Finally, we discuss the reconstitution of membrane proteins using a cell-free synthesis system and the reconstitution and function of multiple membrane proteins.

## 1. Introduction

**Figure 1:**
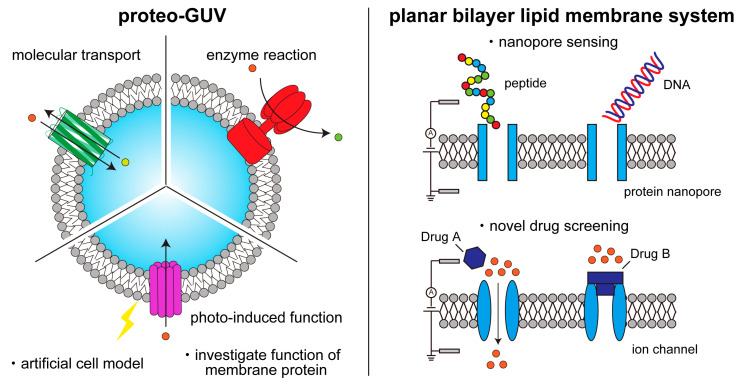
Schematic illustration of functional studies and applications of membrane proteins on artificial lipid membranes.

Membrane proteins are a vital component of the cell membrane, which is composed of a phospholipid bilayer. They play a critical role in various cellular functions, such as molecular transportation, conversion, production, and transduction, including signal transduction, apoptosis, and metabolism [1,2,3,4,5,6,7,8,9,10,11]. Different types of membrane proteins on the cell membrane interact to activate these functions. G protein-coupled receptors (GPCR) and ion channels, which are types of membrane proteins, are important targets for drugs used to treat diseases such as cancer, neurodegenerative disease, metabolic disease and cardiovascular disease [12,13,14,15,16,17]. However, investigating the precise elemental reaction and structure of a membrane protein in complex cell reactions activated by several types of membrane proteins can be challenging. To overcome this issue, methodologies for investigating the function of membrane proteins purified from biological cells have been developed. In the early 1970s, proteoliposomes were first developed by reconstituting the Ca^2+^-dependent ATPase of the sarcoplasmic reticulum into nano-sized liposomes [18,19]. In the late 1980s, the proteoliposomes with K^+^-channels of the sarcoplasmic reticulum were generated using the cell-sized liposomes with diameters of approximately 10 μm, due to the measurement of single ion channel current [20]. Recently, cell-sized proteoliposomes with two or three types of membrane proteins were formed to connect each membrane protein reaction [21,22,23]. Purified membrane proteins have been crystallized to reveal their structures, and they have been reconstituted into artificial cell membranes, such as liposomes, planar lipid bilayers, and bicelles, to observe their functions [24,25,26]. These artificial cell membranes consist of phospholipid bilayers, and the structure and function of the membrane proteins reconstituted into them have been identified using various techniques, such as NMR, fluorescence activated cell sorter, cryo-electron microscopy (cryo-EM), fluorescence spectrophotometry, patch clamp method (for nanopores or ion channels), and optical microscopy, including fluorescence microscopy, and confocal microscopy [25,27,28,29,30,31,32,33,34]. These investigations have not only revealed the functions of membrane proteins but also led to the development of biological sensors using membrane proteins because of their highly specific interactions with ligands and membrane proteins [35,36,37]. Thus, studies of membrane proteins can have a wide range of applications (Figure 1). In this review, we introduce methods for creating liposomes or lipid vesicles, from conventional to recent approaches, as well as techniques for reconstituting membrane proteins into artificial membranes. We also discuss the different types of artificial membranes that can be used to observe the functions of reconstituted membrane proteins, including their structure, number of transmembrane domains, and functional type, as well as the reconstitution of membrane proteins using a cell-free synthesis system and the reconstitution and function of multiple membrane proteins to create artificial cell models. Developing membrane protein reconstitution procedures will contribute greatly to the understanding of membrane protein structure and function, constructing well-defined artificial cell models, and biological sensing with high sensitivity.

## 2. Formation of Cell-Sized Lipid Vesicles

**Figure 2:**
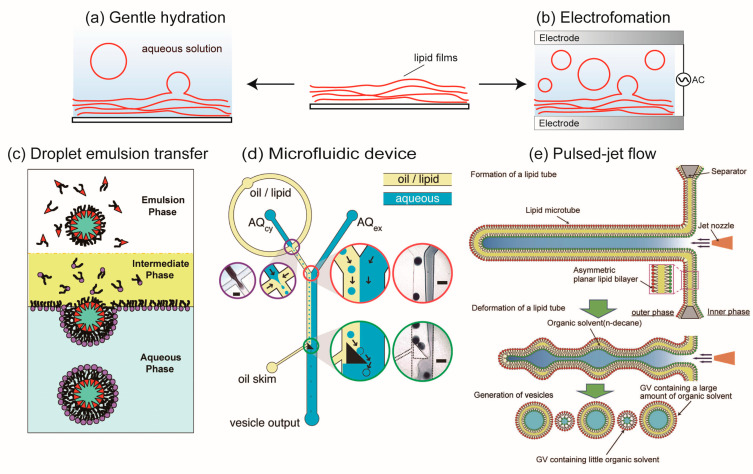
Illustration of giant liposome formation (**a**) Gentle hydration method. (**b**) Electroformation method. (**c**) Droplet emulsion transfer method. Reproduced with permission from * [45]. Copyright (2003) National Academy of Sciences (**d**) Microfluidic devices of cell-sized lipid vesicle formations based on the droplet transfer method * [48]. (**e**) Pulsed-jet flow method * [64]. * Reproduced with permission from.

Membrane proteins are typically reconstituted into artificial cell membranes such as lipid vesicles, liposomes, and bicelles to maintain their structure and function. In this section, we introduce methods for forming lipid vesicles and liposomes, from conventional techniques to recent microfluidic approaches. Liposomes and lipid vesicles, discovered by Bangham in the 1960s, consist of a phospholipid bilayer [38]. Conventional methods for liposome formation include gentle hydration and electroformation (Figure 2a,b) [39,40,41,42,43]. With the gentle hydration method, phospholipids dissolved in chloroform are added to a glass microtube, and the films are formed by evaporating the chloroform under flowing argon or nitrogen gas. The lipid films are then hydrated with an aqueous solution (pure water or buffer solution), forming giant liposomes with diameters of 1–100 μm. Nano-sized liposomes are obtained by vortexing or sonication of the lipid films [1]. With the electroformation method, a lipid film is formed on the surface of an indium tin oxide (ITO)-coated glass. An alternating current (AC) electric field is applied to the lipid films containing a hydrated solution (pure water or a buffer solution with low salt concentration) to enhance lipid hydration. Although the giant liposomes formed by the electroformation method have more unilamellar membranes than those formed by the gentle hydration method, the former has lower liposome production than the latter. Methods have been developed to improve the drawbacks of both the gentle hydration and electroformation methods. These conventional liposome formation methods have been used for studying membrane dynamics, functional properties of membrane proteins, and encapsulation of biological reactions.

The gentle hydration and electroformation methods are limited in their ability to produce liposomes with uniform sizes, in their high encapsulation efficiencies, and in their asymmetric lipid distributions. Microfluidic technologies have been integrated into liposome formation to overcome these limitations and achieve a uniform size and high encapsulation efficiency. The droplet emulsion transfer method, developed by Pautot et al. [44,45], is the basis for many of the liposome formation techniques using microfluidic technologies. The liposome formation procedure with the droplet emulsion transfer method is as follows: First, water-in-oil (w/o) emulsions are generated by vortexing or sonication of buffer solution and phospholipid solution dissolved in an organic solvent such as mineral oil or *n*-decane. The w/o emulsions are then added to the oil phase in a microtube. When the w/o emulsions are transferred to the lipid monolayer between the oil phase and the aqueous phase, liposomes are generated (Figure 2c,d). Asymmetric lipid membranes between the outer leaflet and the inner leaflet can be generated by changing the lipid compositions of the w/o emulsions and the lipid monolayer. By applying the droplet emulsion transfer method, many high-throughput methods for forming liposomes using microfluidic devices have been developed [46,47,48,49,50,51,52,53]. These methods integrate the system of w/o emulsion generation and emulsion transfer from the oil phase to the aqueous phase. Like other methods of liposome formation using microfluidic devices, a new approach has been developed that creates single or multicompartment liposomes by utilizing the de-wetting phenomena of a water-in-oil-in-water double emulsion using a coaxial microcapillary fluidic device [54,55,56,57,58,59,60,61,62].

Another method for forming asymmetric lipid vesicles without an organic solvent (*n*-decane) between the lipid bilayer is by applying a pulsed jet flow against a planar lipid bilayer in a double-well device (called the pulsed-jet flow method) (Figure 2e) [63,64]. The lipid bilayer of liposomes formed by the microfluidic technology method is normally contaminated with an organic solvent, which can cause instability of the liposomes. In contrast, liposomes formed by the pulsed-jet flow method have long-term stability (up to one week). The flip-flop phenomenon and membrane protein reconstitution have been investigated on the asymmetric lipid vesicles formed by this method [64,65]. Modification of the double-well device allows for the formation of nano-sized asymmetric lipid vesicles [66], automatic formation of liposomes [67,68], and the formation of vesicles-in-a-vesicle structures that mimic the asymmetric lipid composition of plasma membranes and intracellular vesicle membranes [69]. Multiple enzyme reactions can be created within the liposomes formed by microfluidic devices [70]. A complex of cell-penetrating peptides and water-soluble proteins can interact with asymmetric lipid vesicles containing negatively charged lipids on the inner leaflet, and the water-soluble proteins can be transferred into the asymmetric lipid vesicles [71].

## 3. Methodology of Reconstitution of Membrane Proteins into Cell-Sized Artificial Membrane

Giant unilamellar vesicles (GUVs) are widely used as models of artificial cells [72,73]. To understand complex biological phenomena in cells, membrane protein function has been studied by reconstituting membrane proteins into GUVs. Various methods have been developed for this, including rehydration, membrane fusion, detergent removal, and direct insertion. In this section, we introduce these methods and provide examples of membrane proteins reconstituted by each method (Table 1) [74,75,76,77,78,79,80,81,82,83,84,85,86,87,88,89,90].

### 3.1. Rehydration Method

**Figure 3:**
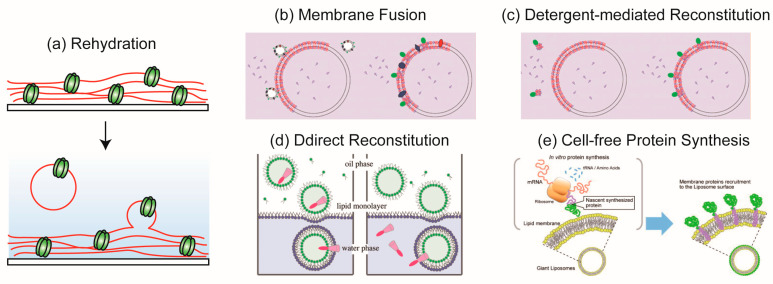
Illustration of proteo-GUVs formation. (**a**) Rehydration method. (**b**,**c**) Detergent-mediated reconstitution and membrane fusion method * [74]. (**d**) Direct reconstitution method * [88]. (**e**) Cell-free protein synthesis with liposome * [91]. * Reproduced with permission from.

In the 1980s, Darszon et al. [91] prepared proteo-GUVs reconstituting bacteriorhodopsin (BR) using the rehydration method (Figure 3a). In this method, membrane proteins are solubilized by mixing micelles of phospholipids and detergent. Proteo-small unilamellar vesicles (proteo-SUVs) are then formed by removing or diluting the detergent below a critical micelle concentration. The solution containing the proteo-SUVs is dried on a glass substrate under nitrogen or argon gas flow to form a thin lipid layer film containing membrane proteins. Proteo-GUVs are generated from the rehydration of the lipid film by adding a buffer solution. Although the rehydration method can prepare proteo-GUVs under physiological conditions, it takes about 1–2 days to form the proteo-GUVs. Girard et al. [92] improved this conventional method by using the electroformation method, which produces higher efficiency in forming proteo-GUVs. In the electroformation method, lipid films containing membrane proteins are prepared on ITO-coated glass, similar to the lipid films produced by the rehydration method. Proteo-GUVs are formed by applying an AC electric field to the hydrated lipid film for approximately 2 h. Proteo-GUVs prepared by the electroformation method have more unilamellar membranes compared to those formed by the hydration method. However, there are some restrictions to using the electroformation method, such as difficulties in preparing GUVs under physiological conditions (containing salt) or containing negatively or positively charged phospholipids. Moreover, the rehydration and electroformation methods require a dehydration step, which may cause the deactivation of membrane proteins due to differences in their structure or transmembrane number.

**Figure 4:**
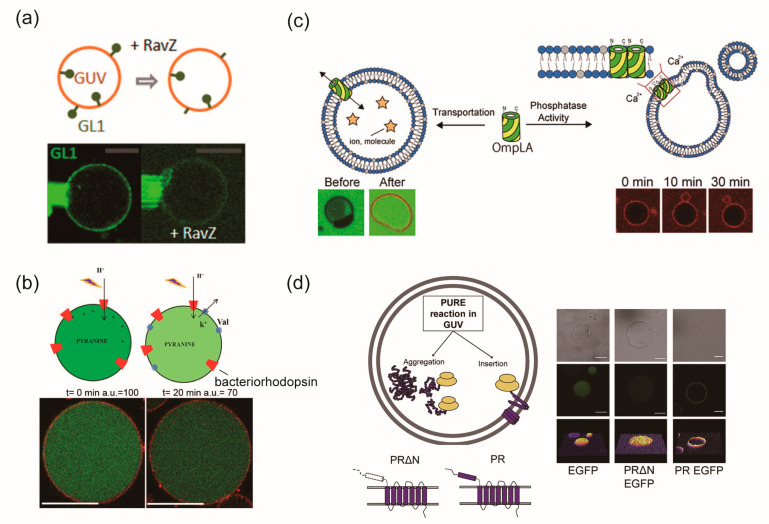
(**a**) Reconstituted protein GL1-Alexa488 is cleaved from the GUV by RavZ, an antiautophagy factor [85] *. (**b**) light-induced proton pumping of BR induced an internal acidification of GUVs [74] *. (**c**) OmpLA transported ions and small molecules. The phospholipase activity caused the budding of small vesicles [77] *. (**d**) N-terminal hydrophobic domain of PR is necessary and sufficient for recruitment of ribosomes to the membrane and membrane insertion of PR [78] *. * Reproduced with permission from.

Motta et al. [85] successfully reconstituted three structurally different membrane proteins, including two transmembrane proteins (Trimeric outer membrane protein, TolC, and the neuronal target soluble N-ethylmaleimide-sensitive factor attachment protein receptor (t-SNARE)) and one lipid-anchored peripheral protein (GABARAP-Like 1(GL1)), using the rehydration method. TolC is an integral membrane protein and part of a heterotrimer that forms a major multidrug efflux pump in *E. Coli*, AcrA-AcrB-TolC. Although its transmembrane part, which has a 12−stranded β−barrel, is correctly folded into the GUV membrane, confirming the multidrug efflux pump function is difficult because the channel must open and close with the help of AcrA and AcrB [85]. The t-SNARE consists of a complex of syntaxin1 and SNAP-25 with a single α−helix transmembrane domain. It plays a role in calcium-dependent synaptic vesicle fusion and forms proximity by complexing with v-SNARE on another vesicle. The soluble domain of v-SNAREs (CDV) specifically binds to t-SNARE-reconstituted proteo-GUV, demonstrating that t-SNAREs are properly folded [85]. GL1, a lipid-anchored peripheral protein, binds to the head group of phosphatidylethanolamine (PE) via an ubiquitin-like reaction involving autophagic enzymes Atg7 and Atg3. GL1 is responsible for the regulation of membrane dynamics in autophagic organelles such as the autophagosome (Figure 4a). Recently, the efficient fabrication of proteo-GUVs using polyvinyl alcohol (PVA) or agarose gel [81] and an electroformation method used Pt wire instead of ITO [93].

### 3.2. Membrane Fusion Method

This method is based on the property of fusion peptides [94] or some detergents [74] to induce the fusion of proteo-SUVs or native membranes (Figure 3b). Kahya et al. [94] prepared proteo-GUV with the BR from *Halobacterium salinarum* using fusion peptides, which are 11-residue amphipathic negatively charged peptides formed into an α−helix and shallowly inserted into the lipid bilayer. The fusion peptide interacts with positively charged SAINT-2 (1-methyl-4,19-*cis*,*cis*-heptatritiaconta-9,28-dienylpyridinium chloride) in the target membrane and promotes membrane fusion [95,96]. The BR is reconstituted into SUV, and the fusion peptide is then coupled to the proteo-SUV via the C-terminal cysteine by overnight conjugation. GUV containing SAINT-2 is prepared by the electroformation method and combined with the peptide-coupled proteo-SUV. The fusion of proteo-SUV and GUV is mediated by the fusion peptide, resulting in GUV being reconstituted with the BR. However, the amount of membrane protein that can be incorporated using this method is limited because the high concentration of the fusion peptide affects the membrane structure and is difficult to remove.

Several detergents, such as Triton X-100, induce fusion in pure lipid large unilamellar vesicles (LUVs), proteoliposomes, and native membranes even at sub-solubilizing concentrations of detergent [74,97,98,99,100]. Dezi [74] developed proteo-GUV by fusing native membrane or proteo-SUV with GUV in the presence of some detergents, which allowed for the reconstitution of BmrC/BmrD, an efflux adenosine triphosphate (ATP)-binding cassette (ABC) transporter from *Bacillus subtilis*. To create proteo-GUVs with various membrane proteins, an inverted inner membrane vesicle (IMV) from *E. coli*, chromatophore purified from photosynthetic bacteria, or proteo-SUV was added to GUV, which was added to the detergent (DOTM) in a sucrose solution. Proteo-GUVs were then formed by incubation without stirring. The amount of detergent used was below the solubilization level of the native vesicles or proteoliposomes [74]. Adding ATP and ethidium bromide (EtBr) to the external aqueous phase of proteo-GUVs containing DNA caused an increase in internal fluorescence due to EtBr binding to the encapsulated DNA. Although this method is required for detergent removal, it is still faster and easier than the membrane fusion method without detergent [82]. Recently, GUVs were used to elucidate the mechanisms by which membrane proteins inhibit the entry of enveloped viruses [87].

### 3.3. Detergent-Mediated Reconstitution Method

Kreir et al. [101] used the detergent-mediated reconstitution method to prepare proteo-GUVs containing the porin outer membrane protein F (OmpF) purified from *E. coli*. In this method, detergent-solubilized OmpF is added to the GUV solution to create proteo-GUVs. After incubating for 1 h at room temperature, bio-beads are added to the proteo-GUVs and then centrifuged to remove the detergent and bio-beads. Using this method, proteo-GUVs containing OmpF and the mechanosensitive channel of large conductance (MscL) were prepared from *E. coli* [101,102]. The functionality of these membrane proteins was confirmed using the single-channel recording method.

Dezi et al. [74] also used the detergent-mediated reconstitution method to prepare proteo-GUVs containing FhuA or BR, and their functionality was confirmed using confocal microscopy (Figure 3c). FhuA, an outer membrane protein of *E. coli*, acts as a receptor for Phages T1, T5, and phi 80, as well as colicin M. To investigate FhuA’s function, proteo-GUV containing soluble YOPRO-1 into the inner phase, which enhances green fluorescence after binding to DNA, was prepared. When T5 phage was added to the proteo-GUV, the fluorescence of YOPRO-1 increased, indicating that loop 8 of FhuA was accessible to T5 phage and that FhuA was inserted through its hydrophobic periplasmic domain in an inside-in orientation. The BR is a seven-transmembrane α-helix light-driven proton pump from *Halobacterium salinarum* that pumps protons from the bacterial cytoplasm to the extracellular space using light energy absorbed by its chromophore retinal. Proton transportation via actinic illumination caused a decrease in pH in GUVs, resulting in a decrease in the fluorescence intensity of the inner phase containing pyranine, a pH-sensitive fluorescent dye (Figure 4b). Dezi et al. [74] reported that the decrease in the fluorescence intensity of the proteo-GUV formed by the detergent-mediated reconstitution method was greater than that prepared by the rehydration method [92]. This means that the amount of proton transportation in the GUVs increased. These results suggest that the BR is not inactivated, and its orientation is aligned. The risk of membrane protein de-activation using this proteo-GUV preparation method is lower than that via the rehydration method because this detergent-mediated reconstitution method does not require dehydration.

### 3.4. Direct Reconstitution Method

In the direct reconstitution method, membrane proteins are spontaneously reconstituted into GUVs from either the inside or outside. Yanagisawa et al. [88] prepared proteo-GUV reconstituting KcsA, a two-transmembrane α-helix from *Streptomyces lividans*, using the direct reconstitution method (Figure 3d). A KcsA monomer was encapsulated in the inner or added to the outer aqueous phase of GUVs prepared by the droplet transfer method. KcsA was spontaneously reconstituted into GUVs from either the inside or outside, and this was accelerated in GUVs containing phosphatidylglycerol (PG) or PE. The internal or external insertion of KcsA leads to an outside-out or inside-out configuration, which retains its hydrophilic cytoplasmic domain on the added aqueous side. Ohnishi and Kamiya [77] prepared proteo-GUV with the outer membrane phospholipase A (OmpLA), a twelve-stranded β-barrel of *E. coli*, by the direct reconstitution method. OmpLA is involved in outer-membrane lipid homeostasis and bacterial virulence. In the presence of phospholipids in the outer leaflet of the lipid bilayer, OmpLA forms homodimers and converts phospholipids to lysophospholipids and fatty acids in a Ca^2+^-dependent manner. When OmpLA was added to the outer aqueous phase of GUVs prepared by the gentle hydration method, it was spontaneously reconstituted into the GUV membrane containing DOPE/DOPG (1:3 weight ratio) or DOPC/DOPG (1:3 molar ratio). In the presence of calcium ions, the OmpLA reconstituted into GUVs exhibited phospholipase activity, and small vesicles budded from the GUVs (Figure 4c).

Direct reconstitution is a simple but challenging method due to the instability of GUVs, and the detergent concentrations used are far below the respective critical micelle concentrations, which can result in protein aggregation [103]. To confirm the production efficiency of proteo-GUV, it is important to search for optimal lipid membranes to reconstitute each membrane protein [77,88].

### 3.5. Reconstitution of Membrane Proteins Using the Cell-Free Protein Synthesis

A bottleneck in the study of membrane proteins is the difficulty of overexpressing them. Although prokaryotic membrane proteins can usually easily be expressed in a host such as *E. coli*, the expression of eukaryotic membrane proteins has proven far more problematic. Unlike soluble proteins, membrane proteins must be targeted and inserted into the membrane as soon as synthesis begins, making overexpression challenging [104]. To address this issue, cell-free synthesis methods using detergents [104], liposomes [105], or microsomes [75] have been investigated via structural and functional studies on membrane proteins. Berrier et al. [104] expressed MscL using the cell-free synthesis of *E. coli* lysate (Rapid Translation System (RTS) [106,107]) in the presence of a detergent. Membrane proteins can be synthesized in the presence of certain detergents, including Triton X-100, Tween 20, Brij 58p, *n*-dodecyl β-D-maltoside, and CHAPS, but inhibited by others, such as *n*-octyl β-D-glucoside and deoxycholate. After purification, the recombinant MscL was added to soybean azolectin, and the detergent was removed using bio-beads. After the removal of the bio-beads, the MscL and azolectin suspension was rehydrated and dried to form proteo-GUV. This MscL channel had the same conductance as those in native *E. coli* membranes or after the reconstitution of purified MscL from *E. coli* [108,109].

Nomura et al. [105] synthesized apo-cytochrome b5 and its fusion proteins using the wheat germ cell-free protein synthesis system [110] in the presence of liposomes prepared by the gentle hydration method (Figure 3e). The synthesized apo-cytochrome b5 was inserted into the liposome and acted as a “hydrophobic tag” to induce b5 fusion hydrophilic proteins (EGFP or dihydrofolate reductase) to the liposome surface.

In addition, R. Eaglesfield et al. [78] reported the mechanism for the reconstitution of proteorhodopsin (PR) to GUVs containing the “protein synthesis using recombinant elements” (PURE) system. The PURE system contains all necessary translation factors, purified to a high specific activity, for efficient protein production [111], and it is a useful tool for investigating the necessary conditions for integrating and functionalizing membrane proteins (Figure 4d).

*E. coli*-based cell-free synthesis systems, such as the RTS and PURE systems, cannot synthesize membrane proteins with post-translational modifications like mammalian proteins without the addition of enzymes. To synthesize post-translationally modified membrane proteins, cell-free synthesis systems extracted from eukaryotic cells, such as rabbit reticulocyte and insect cells, have been used. Fenz et al. [75] synthesized endothelin receptor type-B (ETB) and C-X-C chemokine receptor type 4 (CXCR4), which are classified as GPCR from *Homo sapiens*, using the insect lysate cell-free synthesis system. During protein synthesis, the synthesized ETB or CXCR4 spontaneously reconstituted into microsomal vesicles. DOPC and microsomes containing the synthesized membrane proteins on an ITO-coated glass slide were dried under nitrogen flow. Then, proteo-GUVs containing the microsomal membranes were formed using the electroformation method.

### 3.6. Reconstitution of Membrane Proteins into Cell-Sized Vesicles Composed of Polymer or Proteins

Recently, membrane proteins have been reconstituted into cell-sized vesicles formed from polymer and protein membranes. Cell-sized polymer vesicles have the advantages of higher stability and chemical modification than lipid vesicles. Meier et al. [90,112] generated cell-sized polymer vesicles composed of an amphipathic polymer with poly(dimethylsiloxane) (PDMS, the hydrophobic domain) and poly(2-methyl—oxazoline) (PMOXA, the hydrophilic domain) using a microfluidic device. OmpF, which was reconstituted in this polymer vesicle, transported the enzyme substrates into the polymer vesicle. Marusic et al. [113] reconstituted cytochrome bo3 ubiquinol oxidase into cell-sized polymer vesicles composed of PDMS-*g*-poly(ethylene oxide)(PEO). This polymer vesicle improves the active life of the membrane protein and the resistance of the membranes to free radicals generated by the reaction of this membrane protein. Suzuki and Kamiya [76] reconstituted outer membrane protein G (OmpG) into cell-sized asymmetric vesicles composed of phospholipids on the outer leaflet and amphipathic protein (oleosin) on the inner leaflet. OmpG transports carboxyfluorescein in this asymmetric vesicle. Although studies on these vesicles are at the stage of examining their membrane properties, these vesicles are expected to mimic biological phenomena and can be used in engineering applications, which are difficult to achieve with phospholipid-based liposomes.

## 4. Single-Channel Recording Using Planar Bilayer Lipid Membrane Systems

The function of membrane proteins on cell membranes, such as ion channels and transporters, has been studied using the patch-clamp method. Neher and Sakmann [114] developed the patch-clamp method to measure the function of ion channels and transporters at the single molecule level and in real time. While various patch-clamp methods have been developed for measuring ion currents of ion channels in biological target membranes, artificial planar bilayer lipid membrane (BLM) systems have recently been developed for an engineering-oriented approach [115]. In BLM systems, the target ion channels purified from living cells are reconstituted in the BLM, and the ionic current of the ion channels is measured using a patch clamp amplifier. BLMs allow us to perform functional studies of ion channels in a well-defined environment, such as buffer composition, lipid composition, inhibitor or activator concentration, and membrane potential.

**Figure 5:**
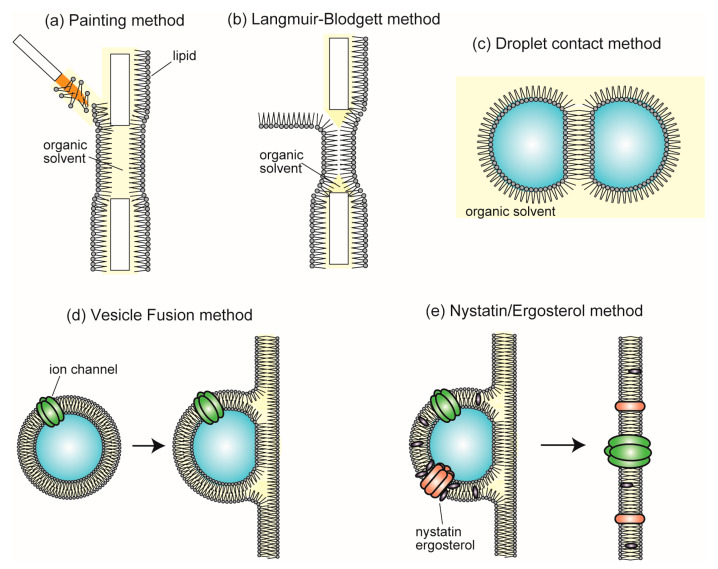
Illustration of BLM formation and the reconstituted ion channel. (**a**) Painting method. (**b**) Langmuir-Blodgett method. (**c**) Droplet contact method. (**d**) Vesicle Fusion method. (**e**) Nystatin/Ergosterol method.

The BLMs (Figure 5a–c) can also be formed by the painting method [116], the Langmuir–Blodgett method [117], or the droplet contact method [63]. In the painting method, a lipid solution (phospholipids dissolved in an organic solvent) is applied across a small aperture that separates two aqueous compartments, forming lipid monolayers. Then, a BLM is formed by the contact of each monolayer after the removal of the organic solvent [116]. This method of BLM formation has improved the Langmuir–Blodgett method by containing a smaller amount of an organic solvent because the presence of the organic solvent in the BLM causes denaturation of the membrane proteins. In the Langmuir–Blodgett method, BLMs are prepared by the apposition of two lipid monolayers spread at the air/water interface. The two monolayers are combined in a small aperture of a Teflon film with a thin layer of *n*-hexadecane or squalene [117]. In the droplet contact method, the lipid bilayer is formed by contacting two water droplets surrounding the lipid monolayer in the double well chip [63]. The painting method or the Langmuir–Blodgett method suffers from fragility and low reproducibility. Therefore, the use of high-throughput systems for pharmaceutical screenings of the BLM formed by these methods is difficult. Conversely, BLMs formed by the droplet contact method can be easily formed by simply dropping each of the solutions.

### 4.1. Reconstitution of Ion Channel into BLMs

The reconstitution of ion channels into BLMs can be done through the direct incorporation method or the vesicle fusion method. In the direct incorporation method, a solution that contains nanopores is added to one or both sides of the BLM’s aqueous compartments, causing spontaneous incorporation. While channel-forming toxins, such as gramicidin A (gA), alamethicin, and α-hemolysin, and bacterial channel proteins such as OmpF can be easily integrated into the BLM [118,119,120,121], reconstituting eukaryotic ion channels such as BEST1 and BK channels is challenging due to their composition of α−helix aggregates in aqueous solution [122,123]. Therefore, the vesicle fusion method is used to reconstitute these ion channels into the BLM [124,125].

In the vesicle fusion method, proteoliposomes or native cell membrane fractions containing target ion channels are added to one or both sides of the BLM device’s aqueous solution (Figure 5d). Fusion between the proteoliposomes or the membrane fractions and the BLM is induced by the following conditions that cause membrane instability: osmotic pressure differences between the solution into the vesicles and the outer buffer solution of the vesicles, the use of negatively charged phospholipids (phosphatidylserine (PS), phosphatidylinositol (PI) or PG) in the BLM, the presence of calcium ions, and the inclusion of PE to create a non-bilayer membrane structure (hexagonal phase) [126]. However, controlling the fusion between the vesicles and the BLM is difficult because this vesicle fusion was spontaneously caused in a time-dependent manner.

Woodbury and Miller [127] proposed the nystatin/ergosterol method to promote and detect the fusion of vesicles with BLMs (Figure 5e). Nystatin is a polyene hydrophobic molecule that forms channels in the presence of high concentrations of sterol (cholesterol or ergosterol). Proteo-SUV containing nystatin and ergosterol can promote fusion with BLMs because the nystatin channel is highly ion-permeable and increases the internal pressure of proteo-SUV [128]. When the proteo-SUV is fused to the BLM without ergosterol, the nystatin-ergosterol complexes dissociate in the BLM. Therefore, the activity of the target ion channels is only observed in the BLM [129].

### 4.2. Protein Nanopore Analysis Using the BLM System

Protein nanopores without gating structures, such as channel-forming toxins and bacterial pore proteins, have been widely investigated because their ion current signal in a BLM is easily obtained via direct incorporation. The OmpG is a 14 β−stranded β−barrel protein with both open and closed conformations owing to its flexible loops in the extracellular region. Chen’s group identified the major loop region responsible for gating in OmpG mutants that modified loop regions using the BLM system [130]. To investigate the gating mechanism of OmpG loops, they analyzed the gating frequency of OmpG mutants with glutamate and arginine residues interacting with the major loop region [131]. Schmitt et al. [132] created OmpG mutants that modified glutamate and arginine residues on the inner surface of the OmpG nanopore and demonstrated that glutamine clusters on the inner surface are associated with cation transport. These functional studies of nanopores using BLM systems have been discussed in greater detail through structural analysis methods, such as NMR [133], X-ray analysis [134], atomic force microscopy (AFM) [135], cryo-EM [136], and molecular dynamics (MD) simulation [137].

Typically, ion current signals of outer membrane proteins are measured from purified proteins expressed by *E. coli*. However, OmpA and OmpG synthesized by the cell-free protein synthesis system can obtain ion current signals similar to those of OmpG expressed by *E. coli* [138]. Recently, the conversion of the pore diameter has been reported by duplicating or replacing one or several β-hairpins of β−barrel nanopores, such as LamB, FhuA, and OmpX [139,140,141]. OmpG mutants with different numbers of β-hairpins showed that the pore diameter and ion current amplitude depend on the number of β-hairpins and the balance of charged amino acids on the inner surface of the OmpG nanopore [142]. Additionally, the R. Kawano group designed de novo nanopores formed by β−barrels and investigated the formation mechanism and ion transport properties of the nanopore using BLM systems and MD simulation [143].

**Figure 6:**
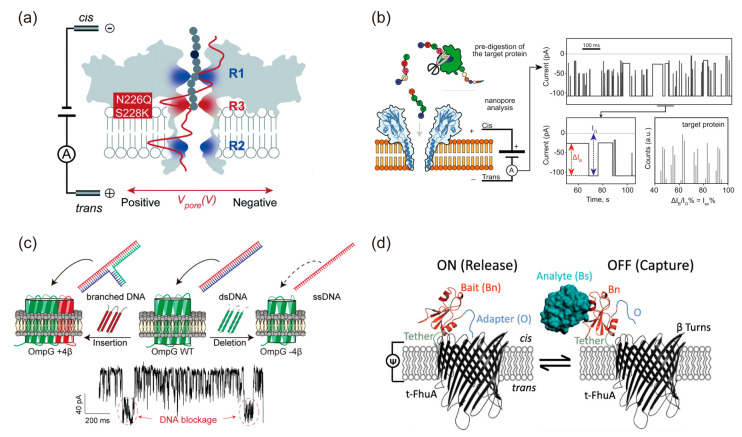
(**a**) Building a new constricted region AeL for heterogeneously charged peptide sensing * [145]. (**b**) Peptides are pre-hydrolyzed by a specific protease and the resulting peptides are measured as they translocate the FraC nanopore [148] *. (**c**) Detection of various structure of DNA depending on the nanopore size using OmpG WT or mutated OmpG with different number of β−hairpins [142] *. (**d**) Detection of the protein analyze outside the FhuA nanopore by means of tethered protein bait [151] *. * Reproduced with permission from.

Furthermore, the technique of single protein nanopore measurement has been applied to the detection of single molecules, such as DNA sequences. In the 1990s, Kasianowicz et al. [144] detected the transport of single-stranded DNA (ssDNA) through the α-hemolysin nanopore at the single-molecule level using BLM systems. This method detected a blockage signal when the α-hemolysin nanopore transported ssDNA. In 2016, the application of this technique was commercialized as a DNA sequencer by Oxford Nanopore Technologies. Single-molecule detection techniques, such as DNA, RNA, and small organic molecules, have been reported using aerolysin (AeL) [145,146,147], fragaceatoxin C (FraC) [148,149], OmpG [142,150], and FhuA [151,152] (Figure 6a–d). Recently, the development of peptide sequencing techniques using protein nanopores, such as AeL, *Mycobacterium smegmatis* porin A (MspA), and proteasome-nanopore, has been reported [153,154,155].

### 4.3. Ion Channel Analysis Using the BLM System

The gating mechanisms and conditions of ion channels, as well as the effects of various reagents (activators or inhibitors), have been studied using the BLM system. One of the most extensively studied ion channels in terms of gating mechanisms and protein conformation is KcsA, a proton-activated channel from *Streptomyces lividans*. The BLM system has been used to study the channel activity of KcsA in response to low pH and phospholipids, such as PG and PI [156,157]. Other ion channels, including prokaryotic channels such as voltage-gated potassium channels (KvAP) from *Aeropyrum pernix* and mechanosensitive channel of small conductance (MscS) from *Pseudomonas aeruginosa* [158,159,160,161,162,163,164], and eukaryotic channels such as large conductance Ca^2+^- and voltage-dependent potassium (BK) channels, cystic fibrosis transmembrane conductance regulator (CFTR), connexin43, and transient receptor potential channel subfamily P member 2 (TRPP2) [122,123,165,166,167,168,169,170,171], have also been analyzed using the BLM system (Table 2).

The BLM system is also useful for screening novel drugs that target eukaryotic ion channels. The human ether-a-go-go-related gene (hERG) channel is a voltage-gated potassium channel in human ventricular myocytes [172]. Drugs designed for completely unrelated targets can result in a potentially fatal arrhythmia by partially or completely blocking the hERG channel [173]. Therefore, investigation into the effects of drugs, including antihistamines, antipsychotics, gastric prokinetics, and even antibiotics, on the hERG channel has been required [174]. Oshima et al. [175] showed that measuring single hERG channels in the presence of the drug can be useful for investigating inhibition mechanisms and designing new drugs without other effects. For systematic screening of ion channel-targeting drugs, a parallel recording method using BLM systems with droplet chamber arrays has been developed [176]. In addition, Kamiya et al. [177] demonstrated that parallel recording of ion channels from biological membrane fractions with each different type of ion channel can be achieved using arrayed BLM systems. Therefore, the BLM system is not only useful for the functional analysis of ion channels, but also for screening new drugs.

## 5. Lipid Vesicles Containing Some Types of Membrane Proteins for Creating Complex Artificial Cell Models

To create artificial cell models that emulate the multiple functions of living cells, various membrane protein functions, such as molecular transportation, molecular conversion, and molecular production, must be reconstituted into lipid vesicles. Additionally, fluorescent probes or biomolecules such as DNA, water-soluble proteins, in vitro transcription-translation systems, and ions have been encapsulated into lipid vesicles. These complex artificial cell models, based on a bottom-up approach, have been constructed worldwide. In this section, we introduce lipid vesicles containing two or more types of membrane proteins.

**Figure 7:**
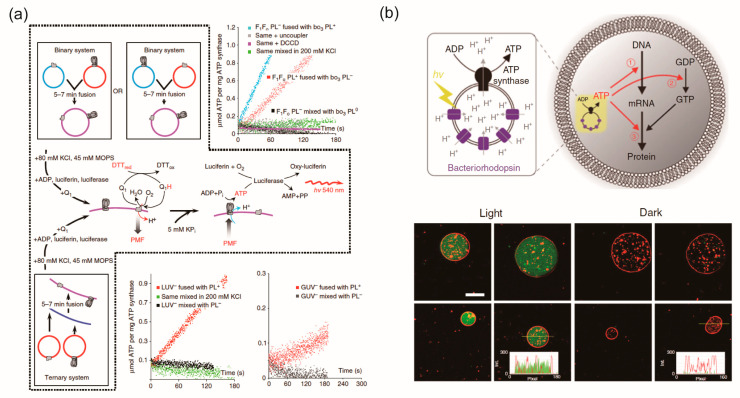
(**a**) Modular assembly of a functional electron transport chain by delivering ATP-synthase and bo3-oxidase into target membrane [21] *. (**b**) Schematic illustration of the artificial photosynthetic cell encapsulating artificial organelle containing BR and ATP synthase. Green fluorescent protein was synthesized inside GUV driven by light [22] *. * Reproduced with permission from.

Ishmukhametov et al. [21] developed lipid vesicles reconstituted with ATP synthase and bo3-oxidase through fusion between cationic and anionic lipid vesicles containing either ATP-synthase or bo3-oxidase (Figure 7a). This created a molecular bio-synthetic network linking the primary (proton motive force, PMF) and secondary ATP forms of biological free energy. Detergent removal using a Sephadex G-50 column reconstituted ATP synthase and bo3-oxidase into the lipid vesicles. When reduced dithiothreitol and Coenzyme Q_1_ were added to the outer solution of the lipid vesicles, PMF was generated toward the inner solution because protons were also transferred to the inner solution. With PMF and KPi added to the inner solution, ATP was synthesized from ADP and pyrophosphate by ATP synthase in the outer solution. The ATP production of this molecular biosynthetic network was detected by luminescence using the luciferin-luciferase system.

Berhanu et al. [22] developed nano-sized lipid vesicles containing ATP synthase and BR for synthesizing proteins of an in vitro transcription–translation system using ATP produced from the nano-sized lipid vesicles by light (Figure 7b). Detergent removal using bio-beads reconstituted ATP synthase and BR into the nano-sized lipid vesicles, which were then encapsulated into cell-sized lipid vesicles along with the in vitro transcription–translation system. When light was irradiated to the cell-sized lipid vesicles, BR on the nano-sized lipid vesicle membranes transported protons into the nano-sized lipid vesicles. By increasing the proton concentration into the inner solution of the nano-sized lipid vesicles, ATP-synthase converted ADP to ATP in the outer solution of the nano-sized lipid vesicles (in other words, into the inner solution of the cell-sized lipid vesicles). The produced ATP was consumed as substrates for messenger RNA, energy for phosphorylation of guanosine diphosphate, or energy for aminoacylation of transfer RNA.

Lee et al. [23] developed nano-sized lipid vesicles containing ATP synthase, PR, and plant-derived photosystem II (PSII) for controlling ATP production by changing the proton concentration and switching PR or PSII activity using red or green light. ATP synthase, PR, and PSII were reconstituted in the nano-sized lipid vesicles using a detergent-mediated method. The activation mechanism of the nano-sized lipid vesicles containing ATP-synthase, PR, and PSII was as follows: red light irradiated to the nano-sized lipid vesicles generated protons into the inner solution of the nano-sized lipid vesicles using PSII, which subsequently increased the proton concentration and caused ATP synthase to convert ADP to ATP in the outer solution. Conversely, green light caused protons to move from the inner to the outer solution of the nano-sized lipid vesicles, decreasing the proton concentration and halting ATP synthesis. Cell-sized lipid vesicles containing three types of membrane protein-reconstituted nano-sized lipid vesicles, ionophore, and actin experienced actin polymerizations and morphological changes depending on the ATP concentration generated from the nano-sized lipid vesicles.

## 6. Conclusions and Future Direction

In this review, methodologies for the reconstitution of membrane proteins into GUVs or BLMs are introduced. The functions of various membrane proteins in GUVs and BLMs have been investigated with the development of reconstitution technologies. Therefore, the reconstitution of membrane proteins will become a powerful tool for investigating precise elemental reactions or responses to reagents. The orientation of membrane proteins is responsible for regulating membrane protein functions, such as ion transportation, signal transduction, and substance conversion. Currently, it is difficult to completely control the orientation of membrane proteins. Therefore, novel technologies for orientation control are required to reconstitute membrane proteins in GUVs. Moreover, the relationship between the number of reconstituted membrane proteins and the number of functionalized membrane proteins is unclear. Although it is difficult to reveal a unified theory to understand this relationship, the number of functionalized membrane proteins on liposomes may be estimated from the relationships of some membrane proteins. Understanding this relationship will lead to the optimization of the reaction of membrane proteins on liposomes. We believe that not all types of membrane proteins in artificial lipid membranes have a function because no proteoliposome formation method applies to all types of membrane proteins. Therefore, optimization of the purification and reconstitution methods for membrane proteins, lipid compositions (neutral, negatively, or positively charged phospholipids, and phospholipids with saturated or unsaturated alkyl chains), and buffer compositions (salt concentration or pH) are required for the reconstitution of functionalized membrane proteins into artificial lipid membranes.

The advantages of the reconstitution of membrane proteins into GUVs include the reconstitution and functionalization of membrane proteins across various types and species on GUVs. The cascade reactions, as designed, construct a connection with membrane proteins and other biomolecules, such as an electronic circuit. Well-defined artificial cell models with communication between cells, production of materials, fission, and growth will be constructed by reconstitution technologies of the membrane proteins combined with the GUV formation technologies of microfluidic devices and other technologies. Therefore, membrane protein reconstitution will play an important role in the elucidation of the functional mechanisms and structures of membrane proteins and for drug discoveries as fundamental studies, and in the construction of artificial cell models, including the modulation of living cell functions and energy production, and biological sensors, including diagnosis and environmental sensing.

## Figures and Tables

**Table 1:** Selected examples of membrane proteins reconstitution in GUVs.

Protein	Organism	Type	TM Region	Complex	TM Number	Method	Membrane Composition	Ref.
BmrC/BmrD	*Bacillus subtilis*	ABC transporter	α−helix	BmrC/BmrD	12	detergent mediate reconstitution	DPhPC + DOPC/DOPE or DOPC/Sph/chol	[74]
BR	*Halophilic archaea*	proton pump	α−helix	−	7	fusion	EPC:EPA (9:1 [mol])	[74]
CXCR4	*Homo sapiens*	GPCR signaling protein	α−helix	homodimer	14 (7 × 2)	fusion	DOPC	[75]
EmrE	*Escherichia coli*	multidrug transporter	α−helix	homodimer	8 (4 × 2)	direct reconstitution	POPC	[83]
ETB	*Homo sapiens*	GPCR signaling protein	α−helix	−	7	fusion	DOPC	[75]
F_1_F_0_-ATP synthase	*Escherichia coli*	ATP production	α−helix	F_1_–F_0_	28	rehydration	*E. coli* total lipid extract	[84]
FhuA	*Escherichia coli*	ferrichrome-iron receptor	β−strand	−	22	fusion	EPC:Tx-DHPE (99.5:0.5)	[74]
GL1	*Homo sapiens*	γ-aminobutyric acid receptor	α−helix	−	anchored	rehydration	DOPE:DOPC:DOPE-Atto647 (30:69.5:0.5)	[85]
GLUT1	*Homo sapiens*	glucose transporter	α−helix	−	12	fusion	DOPC:DMPE-RhB:Biotylated PE (99.7:0.2:0.1)	[86]
IFITM3	*Homo sapiens*	enveloped virus inhibitor	α−helix	−	1	detergent mediate reconstitution	POPC:cholesterol:Liss-Rho-PE (99:0.5:0.5 [mol])	[87]
KcsA	*Streptomyces lividans*	potassium channels	α−helix	homotetramer	8 (2 × 4)	direct reconstitution	DOPG or DOPE	[88]
KvAP	*Aeropyrum pernix*	voltage-gated potassium channels	α−helix	heteromer or homotetramer	8 (2 × 4)	rehydration	DPhPC or EPC:EPA (9:1)	[89]
OmpF	*Escherichia coli*	porin	β−strand	−	16	direct reconstitution	PDMS26-b-PMOXA9	[90]
OmpG	*Escherichia coli*	porin	β−strand	−	14	direct reconstitution	Outer membrane: DOPC Inner membrane: oleosin	[76]
OmpLA	*Escherichia coli*	phospholipase	β−strand	homodimer	24 (12 × 2)	direct reconstitution	DOPC:DOPG (1:3)	[77]
PR	SAR86 group of marine γ-proteobacteria	proton transport	α−helix	−	7	direct reconstitution	POPC	[78]
RC	*Rhodobacter sphaeroides*	electron transport	α−helix	−	10	detergent mediate reconstitution	POPC:POPG (9:1 [mol])	[79]
SLO	*Streptococcus pyogenes*	toxin	α−helix	homo 36~40 mer	36~40	rehydration	POPC, DOPC, SOPC, POPG	[80]
TmrAB	*thermus thermophilus*	ABC transporter	α−helix	−	6 × 2	rehydration	POPC:POPG:POPE: biotinylated-DOPE (40:30:29:1 [mol])	[81]
TolC	*Escherichia coli*	expulsion of diverse molecules from the cell	β−strand	homotrimer	12 (4 × 3)	rehydration	DOPC:DOPS:Atto647-DOPE (91.2:8:0.8)	[85]
t-SNARE	*Homo sapiens*	endocytosis/exocytosis	α−helix	Syntaxin -SNAP25	1	rehydration	DOPC:DOPS:Atto647-DOPE (91.2:8:0.8)	[85]
VDAC1	*Homo sapiens*	voltage dependent anion channel	β−strand	−	19	fusion	soybean polar extract: cholesterol (9:1)	[82]

**Table 2:** Selected examples of ion channels analysis using BLM system.

Ion Channels	Organism	Type	TM Region	Complex	TM Number	Membrane Composition	Ref.
FocA	*Salmonella typhimurium*	formate channel	α−helix	homohexamer	30 (5 × 6)	*E. coli* polar lipid extract	[158]
KcsA	*Streptomyces lividans*	K^+^ channel	α−helix	homotetramer	8 (2 × 4)	L-α-lecithin	[159]
KvAP	*Aeropyrum pernix*	K^+^ channel	α−helix	homotetrameric	8 (2 × 4)	cardiolipin:cholesterol (6:1)	[160]
MscL	*Escherichia coli*	mechanosensitive channel	α−helix	homopentamer	10 (2 × 5)	DPhPC	[161]
MscS	*Pseudomonas aeruginosa*	mechanosensitive channel	α−helix	homoheptamer	31 (3 × 7)	*E. coli* polar lipid extract	[162]
MVP	*Methanococcus jannaschii*	voltage-gated K^+^ channel	α−helix	homotetramer	24 (6 × 4)	POPE:POPG (3:1)	[159]
Nav	*Bacilllus halodurans*	voltage-gated Na^+^ channel	α−helix	homotetramer	24 (6 × 4)	POPE:POPG (3:1 [wt]/[wt])	[163]
NirC	*Salmonella typhimurium*	potential nitrite transporter	α−helix	homohexamer	30 (5 × 6)	*E. coli* polar lipid extract	[164]
BEST1	*Homo sapiens*	Ca^2+^ activated chloride channel	α−helix	homopentamer	20 (4 × 5)	POPE:POPG (3:1 [wt/wt])	[122]
BK	*Homo sapiens*	Ca^2+^ -and voltage-dependent potassium channel	α−helix	Homotetramer	8 (2 × 4)	POPE:POPS (1:1)	[123]
CFTR	*Homo sapiens*	chloride channel	α−helix	−	12	POPE:POPS (2:1)	[165]
connexin43	*Homo sapiens*	gap junction channel	α−helix	homodimer	24 (4 × 6)	POPC	[166]
Kir3.4	*Mus musculus*	cholesterol sensitive potassium channel	α−helix	homotetramer	8 (2 × 4)	PE:POPS (1:1)	[167]
P2X7R	*Homo sapiens*	ATP-gated cation-selective channel	α−helix	homotrimer	6 (2 × 3)	*E. coli* polar lipid extract	[168]
RYR2	*Homo sapiens*	Ca^2+^ release channel	α−helix	homotetramer	24 (6 × 4)	DOPE:DOPS (3:1)	[169]
Sec 61	*Homo sapiens*	dynamic polypeptide-conducting channel	α−helix	3-domain α/β/γ	12	L-α-lecithin	[170]
TRPP2	*Homo sapiens*	Ca^2+^ permeable nonselective cation channel	α−helix	homotetramer	24 (6 × 4)	POPC:POPE (7:3)	[171]

## Data Availability

Not applicable.
